# Towards the Automatic Classification of Avian Flight Calls for Bioacoustic Monitoring

**DOI:** 10.1371/journal.pone.0166866

**Published:** 2016-11-23

**Authors:** Justin Salamon, Juan Pablo Bello, Andrew Farnsworth, Matt Robbins, Sara Keen, Holger Klinck, Steve Kelling

**Affiliations:** 1 Music and Audio Research Laboratory, New York University, New York, NY, United States of America; 2 Center for Urban Science and Progress, New York University, New York, NY, United States of America; 3 Cornell Lab of Ornithology, Cornell University, Ithaca, NY, United States of America; Virginia Commonwealth University, UNITED STATES

## Abstract

Automatic classification of animal vocalizations has great potential to enhance the monitoring of species movements and behaviors. This is particularly true for monitoring nocturnal bird migration, where automated classification of migrants’ flight calls could yield new biological insights and conservation applications for birds that vocalize during migration. In this paper we investigate the automatic classification of bird species from flight calls, and in particular the relationship between two different problem formulations commonly found in the literature: classifying a short clip containing one of a fixed set of known species (N-class problem) and the continuous monitoring problem, the latter of which is relevant to migration monitoring. We implemented a state-of-the-art audio classification model based on unsupervised feature learning and evaluated it on three novel datasets, one for studying the N-class problem including over 5000 flight calls from 43 different species, and two realistic datasets for studying the monitoring scenario comprising hundreds of thousands of audio clips that were compiled by means of remote acoustic sensors deployed in the field during two migration seasons. We show that the model achieves high accuracy when classifying a clip to one of N known species, even for a large number of species. In contrast, the model does not perform as well in the continuous monitoring case. Through a detailed error analysis (that included full expert review of false positives and negatives) we show the model is confounded by varying background noise conditions and previously unseen vocalizations. We also show that the model needs to be parameterized and benchmarked differently for the continuous monitoring scenario. Finally, we show that despite the reduced performance, given the right conditions the model can still characterize the migration pattern of a specific species. The paper concludes with directions for future research.

## Introduction

Many organisms, particularly birds, vocalize frequently, and these signals are the means by which detection and classification occurs. These signals may represent the best means of surveying individuals or populations. However, much biodiversity monitoring has been performed by expert humans, who typically rely on auditory cues to detect and classify bird species, because replicating and automating humans’ abilities have proven very challenging to the extent that automated systems cannot routinely replace humans in conducting biodiversity surveys for birds. Yet, a number of factors constrain human monitoring, including variability in observer skills in detection and classification [1–3], temporal mismatches in observer effort and biological phenomena [4, 5], and spatial mismatches in observer coverage and biological distributions [6, 7].

Automated classification of organisms to species based on their vocalizations would contribute tremendously to abilities to monitor biodiversity, with a wide range of applications in the field of ecology. Acoustic monitoring provides information about biodiversity and changes in its spatial and temporal distribution [8], and, with coordinated application with existing technologies, about migrating species in areas where potential hazards exist, such as collisions with buildings, planes, communications towers, and wind turbines. Acoustic monitoring can provide the unique benefits of knowing species composition wherever and whenever microphones and recorders are deployed. This includes long term recording from remote locations and continuous recording for periods when observers are not active. One particular application where such automation would provide unique and complementary information is in monitoring nocturnal bird migration.

Current tools to study nocturnal bird migration rely on several sources of information to produce details of bird movements. These sources include weather surveillance radar, which provides insight into the density, direction, and speed of bird movements but little to no information about the species actually migrating [9], tracking devices that provide information about the location and activity of individuals but little information about population behaviors, marking (e.g. banding, ringing, or color marking) and later recapturing individuals, with similar constraints, stable isotope tracking of the changes in key elements to define movements of birds [10, 11], and crowdsourced human observations, made almost exclusively during daytime hours and of limited use for studying nocturnal migratory flights other than by proxy [12]. Automatic bioacoustic monitoring and analysis is a complementary solution that could be scalable and produce species-specific information otherwise impossible to obtain from any of the previously mentioned methods.

Among an increasingly important array of bioacoustic tools for conservation science [13] that describe presence, abundance, and behavior of vocal species, there is a significant body of research on automatic species classification (e.g. [14–21]). See [22] for a detailed survey of automatic birdsong recognition. Of these, a number of systems target acoustic monitoring [16, 18, 19, 21, 23], some focusing specifically on bird flight calls [24–26]. Such systems can be broadly divided into two groups: those designed to distinguish among a predefined set of known species [14, 15, 17, 20, 27], and those designed to identify a specific species in a continuous audio stream [16, 18, 19, 21, 23]. The former is posed as an N-class classification problem: given a collection of sound clips where each clip contains the vocalization of one of N possible species, train a model to correctly classify the species. Recent studies have obtained highly promising results for birdsong classification under this scenario, in particular through the employment of feature learning techniques [20]. However, the N-class scenario is not necessarily a good match for bioacoustic monitoring, as it abstracts away the primary, and critical, challenge of having to detect vocalizations in a continuous stream of what is mostly non-relevant geophony (e.g. wind, water), biophony (e.g. insects, frogs) and anthrophony (e.g. speech, transportation). Furthermore, many of the datasets used in such studies were recorded under relatively homogeneous conditions, featuring bird vocalizations in the foreground with relatively little background noise. These are ideal but unrealistic conditions for in-the-field acoustic monitoring systems.

The latter set of systems pose the problem as one of event detection and classification. For nocturnal migration monitoring, the first generation of systems captured audio data via recording stations [28, 29], but for many years the identification of species was performed via aural and visual inspection of the recording and spectrogram, respectively, by experts [30]. Automatic classifiers later replaced such inspection, however, in many cases these automated systems were carefully crafted by exploiting species-specific characteristics and building them into the model through custom preprocessing or feature design [24] or by comparing to a set of preselected and preprocessed spectral templates [25, 26]. A primary disadvantage of such systems is that they require complete (and manual) readjustment to work for a different species. Furthermore, many of the aforementioned studies make use of relatively small datasets, usually on the order of a few hundred audio clips, and it is thus unclear how well they would perform at a larger scale. More recently, a number of approaches have been proposed that employ more advanced and generalizable machine learning techniques that can be easily adapted to multiple species [19, 31, 32]. These studies were focused on bird song (and marine mammals) however, not flight calls. Among other differences from vocalizations analyzed in previous studies, flight calls are primarily single note vocalizations that are less than 200 ms long, whereas most songs contain several types of notes and may vary from seconds to minutes in duration.

In this paper we investigate the automatic classification of bird species from flight calls, and in particular the relationship between the two problem formulations described above: the N-class problem, and the continuous monitoring problem which as noted earlier is the scenario relevant to nocturnal migration monitoring. The study has two principle goals:

1. To contrast the performance of the same classification architecture on the N-class problem and the continuous acoustic monitoring problem.
2. To study the performance and limitations of the classification architecture under a real-world scenario: acoustic monitoring where the test data is open-set (unconstrained in terms of possible classes) and whose class distribution is unknown a-priori.

To this end, we implemented a state-of-the-art audio classification model based on unsupervised feature learning that has been applied successfully to music information retrieval [33], urban sound classification [34, 35] and birdsong classification [20]. To the best of our knowledge this is the first time a feature-learning-based audio classification technique has been applied to flight call classification. To ensure our experiments are representative of real-world scenarios, we constructed novel datasets for evaluating the aforementioned approach. For the N-class problem, we compiled a dataset of over 5000 flight calls from 43 different bird species. For the monitoring problem, we collected a large amount of audio data using remote acoustic sensors deployed in the field during two migration seasons. The data collected contains realistic challenges such as varied background and foreground noise and a significant unbalance between the size of the target class (clips containing the flight call of a target species) and the negative class (clips containing all other flight calls and geophonic, biophonic and anthrophonic noise), which is considerably larger. Through a series of experiments we show that:

1. High accuracy is achievable for flight call classification under the N-class scenario even for a large number of species.
2. The classification system needs to be optimized and benchmarked differently for each of the two scenarios (N-class and acoustic monitoring).
3. The system is not as robust for in-the-field event classification (acoustic monitoring). Still, given the right conditions the model can successfully characterize the nocturnal migration pattern of a specific species.

The remainder of this paper is organized as follows: in the following section (Methods), we describe the feature-learning-based classification model implemented for this study and the metrics used to evaluate it. This is followed by a detailed description of the datasets used in this study, including how they were compiled and their key characteristics (Data). Next we present and discuss the results obtained for the various experiments conducted in this study (Results and Discussion). We examine the results for the N-class problem, and then contrast them with those obtained in the acoustic monitoring scenario for two different target species. The final section of this paper (Conclusions and Future Work) summarizes the results presented in the paper and the key discussion points, and provides some proposed directions for future research.

## Methods

**Fig 1:**
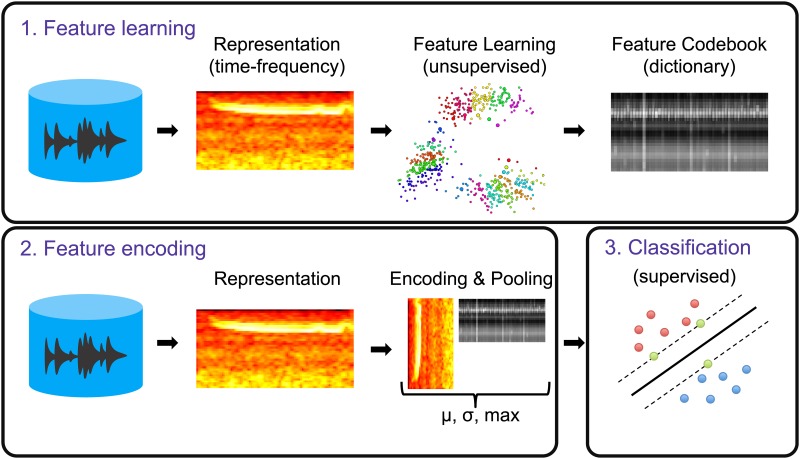
Block diagram of the classification framework comprising 3 main blocks: (1) Feature learning (learn a codebook from the train data), (2) Feature encoding (use the learned codebook to encode the train and test data), (3) Classification (use the encoded train and test data to fit and evaluate a discriminative classifier).

The construction of our classification model can be divided into three main blocks: (1) feature learning, (2) feature encoding and (3) classification (Fig 1). The goal of the first block was to learn a dictionary (or codebook) of representative bases (or codewords) from the training data by means of an unsupervised data-driven process. In the second block, the learned dictionary was used to encode the samples of the dataset into feature vectors. Finally, in the third block these feature vectors were used to train (and test) a discriminative classifier. The details of each of the three blocks are provided below.

### Feature learning

The goal of this block was to learn a dictionary of representative (and discriminative) codewords directly from the audio signal in a data-driven fashion. Although there have been some recent advancements in learning audio representations directly from the time-domain signal [36, 37], such techniques are computationally complex, require thousands of hours of audio data for training, and still only perform comparably to feature learning from a time-frequency (e.g. spectral) representation which can be performed more efficiently using a smaller model. Consequently, for this work we extracted log-scaled mel-spectrograms with 40 components between 2000–11025 Hz. The flight calls of the species studied in this work all fall inside this frequency range. Since flight calls are short (compared to e.g. birdsong), on the range of 50–150 ms in duration with fast frequency modulations, it was important that our processing pipeline be parametrized so that it could capture the fine temporal structure of these calls. After some initial experimentation, we settled on an analysis window (Hann) of 11.6 ms (256 samples at a sampling rate of 22050 Hz), and a hop size (the time interval between consecutive analysis windows) of 1.45 ms (32 samples). In [34] we showed that feature learning is more effective (compared to using standard audio features based on Mel-Frequency Cepstral Coefficients (MFCC) [38]) when the learning is performed jointly on groups of frames (also referred to as “shingles” or 2D time-frequency patches) since the learned features can capture spectro-temporal shapes that are representative of the different classes of interest. Following this result, in this study we also used 2D time-frequency patches (henceforth TF-patches). The duration of the TF-patches used for learning was determined via cross-validation on the training set, the details of which shall be explained later on. Furthermore, decorrelating the input dimensions prior to feature learning improves the discriminative power of the learned features [39], and so before passing the 2D log-mel-spectrogram patches to the learning stage we applied Principal Component Analysis (PCA [40]) to the entire training set and scaled each component such that the resulting feature dimensions are uncorrelated and have unit variances (PCA whitening). Following [33] we kept enough components to explain 99% of the variance in the data. The PCA parameters learned from the training data were also used for encoding as detailed in the Feature encoding section below.

To learn the codebook we used the *spherical k-means* algorithm [39]. Unlike the traditional k-means clustering algorithm [41], the centroids are constrained to have unit L2 norm (they must lie on the unit sphere, preventing them from becoming arbitrarily large or small), and represent the distribution of meaningful directions in the data. Compared to standard k-means, spherical k-means is less susceptible to events carrying a significant amount of the total energy of the signal (e.g. background noise) dominating the codebook. The algorithm is efficient and highly scalable, and it has been shown that the resulting set of vectors (the centroids) can be used as bases (a dictionary) for mapping new data into a feature space which reflects the discovered regularities [20, 39, 42]. The algorithm is competitive with more complex (and consequently slower) techniques such as sparse coding, and has been used successfully to learn features from audio for music [33], birdsong [20] and urban sound classification [34, 35]. After applying this clustering to our training data, we used the resulting cluster centroids as the codewords of our learned dictionary. Note that we used the algorithm as a feature learning technique, and not for the purpose of clustering the data into the actual number of classes they are comprised of. As such, we used the algorithm to learn an over-complete codebook, meaning the values tested for *k* (described in the Evaluation section) were much larger than the number of classes (in our case, species) present in the data.

The clustering was performed as follows: let us represent our data as a matrix $X\in R^{n\times m}$, where every column vector $x^{\left(i\right)}\in R^{n}$ is the feature vector for a single sample (in our case a whitened log-mel-spectrogram TF-patch), *n* is the number of dimensions in each feature vector and *i* = 1…*m* where *m* is the total number of samples. We use *s*^(*i*)^ to denote the code vector for sample *i* which stores a binary assignment value for each of our *k* clusters (with ||*s*^(*i*)^||_0_ ≤ 1, ∀*i*, i.e. only one element in *s*^(*i*)^ can be non-zero). For convenience, let *S* be the matrix whose columns are *s*^(*i*)^. Finally, let $D\in R^{n\times k}$ represent our codebook of *k* vectors (means). Then, the spherical k-means algorithm can be implemented by looping over the following three equations until convergence:

$$s_{j}^{\left(i\right)}:=\left\{\begin{array}{c} D^{(j)⊤}x^{\left(i\right)}\;\text{if}\;j==\arg\;\max_{l}{\left|D^{(l)⊤}x^{\left(i\right)}\right|}_{\forall j,i}\\ 0\;\;\;\;\;\;\text{otherwise.} \end{array}\right.$$ (1)

$$D:=XS^{⊤}+D$$ (2)

$$D^{\left(j\right)}:=D^{\left(j\right)}/\left|\right|D^{\left(j\right)}{\left|\right|}_{2}\forall j$$ (3)

where ⊤ indicates matrix transposition. In Eq (1) we assign samples to centroids, in Eq (2) we update the centroids, and finally in Eq (3) we normalize the centroids to have unit L2 norm. Before running the algorithm we randomly initialized the centroids in the codebook D from a Normal distribution and normalized them as in Eq (3). For further details about the algorithm the reader is referred to [39].

### Feature encoding

The spherical k-means algorithm produces a codebook matrix with *k* columns, where each column represents a learned codeword (centroid). We used the codebook to encode every sample in our dataset into a feature vector. Given an audio recording, we extracted the log-mel-spectrogram as before, sliced it into a series of overlapping TF-patches and applied PCA to each patch using the same PCA parameters that were learned in the previous block when learning the codebook (i.e. the PCA parameters can be considered part of the model training process: they were learned from the training data and kept fixed for encoding the test data). The result can be represented as a matrix $M\in R^{n\times m}$ where each column represents one (flattened) TF-patch after PCA whitening (similar to *X* from the previous block except M contains TF-patches from a single audio recording whereas *X* comprised the TF-patches of the entire training set). Encoding was performed by taking the matrix product between $M^{⊤}$ and the codebook D:

$$F=M^{⊤}D\in R^{m\times k}.$$ (4)

The resulting matrix F has *k* columns and a row for each row in the input matrix, where the number of rows *m* depends on the duration of the recording. Every column *i* (*i* = 1…*k*) in the encoded matrix F can be viewed as a time-series whose values represent the match scores between the TF-patches of the input recording and the *i*^*th*^ codeword in the codebook: when a TF-patch is similar to the codeword the value in the time-series will be higher, and when it is dissimilar the value will be lower. The encoded matrix F cannot be used directly as input features to the classifier since for each recording the matrix can have a different number of rows (depending on the duration of the recording), whereas we require a feature vector of fixed dimensionality. To obtain a consistent dimensionality for every clip in the dataset regardless of its duration (for the N-class scenario we used clips of varying duration, and for the acoustic monitoring scenario the continuous signal was segmented into short clips of equal duration), the matrix was summarized over the time dimension by computing a number of summary statistics (such as the mean, standard deviation, and maximum) from each column of F, resulting in a feature vector of size *k* * *l* where *l* is the number of summary statistics computed. Both *k* and the specific set of summary statistics to be computed were treated as model hyper-parameters (i.e. model parameters that must be specified before the model is trained as opposed to the model parameters that are learned during training) and determined via cross validation. Further details about the specific values of *k* and summary statistics used in the experiments are provided in the Evaluation section.

### Classification

The output of the encoding, a feature vector of size *k* * *l* for every sample in the dataset, can be used to train (and test) a discriminative classifier of our choice. Following the results of [20, 34], we experimented with two classification algorithms: a Random Forest classifier [43] and a Support Vector Machine (SVM) classifier with a radial basis function kernel [44]. The choice of classifier (and classifier hyper-parameters) was treated as another hyper-parameter to be determined via cross-validation on the training set. Since in all experiments conducted in this study the SVM was always chosen over the Random Forest, for the sake of clarity and conciseness we shall only discuss the results obtained using the SVM classifier. Prior to training the classifier the features were standardized across samples. We used the *Essentia* library [45] for computing the log-mel-spectrograms and the *scikit-learn* library [46] for training the classifier, both of which are open source.

To ensure the model generalizes as best as possible to the test data we would like the distribution of classes in the training data to be as representative (similar) as possible to the distribution of classes in the test data. In the case of the controlled N-class problem, we achieved this by using stratified train/test splits with 5-fold cross-validation (further details are provided in the Evaluation section). For the acoustic monitoring problem, however, we sought to simulate a real-world scenario where we would have to train the model with the data collected and labeled to date (in this study represented by the data collected during fall 2014, as detailed in the Data section) and run it on data that would be collected during future migration seasons (in this study represented by the data collected during spring 2015, as detailed in the Data section), the distribution of which, therefore, would be unknown at the time of training. The best approximation we can obtain of the test data distribution under this scenario (in the absence of additional (meta)data such as eBird species distributions [8]) is given by the unweighted class distribution of the training data. Consequently, for the acoustic monitoring scenario we trained the model using the training data with equal weighting for every datum. For further discussion of the challenges presented by this type of open-set problem (i.e. where the distribution of classes in the test data is not known a-priori) in the context of bioacoustics see [47].

We were particularly interested in this scenario since deploying a classification system such as the one proposed in this study for migration monitoring at a large scale necessarily means the model will have to cope with new environments (locations/seasons) that are not necessarily well represented by the training data (or not at all). How well our model generalized to such environments (and under what scenarios it failed to generalize) was one of the key questions of this study.

### Evaluation

**Baseline:** To assess how well the proposed model performed on the N-class problem compared to a standard audio classification approach which does not employ feature learning (a baseline model), we also implemented and evaluated a model which extracts 25 Mel-Frequency Cepstral Coefficients (MFCC [38]) per-frame using a Hann window of 11.6 ms and a hop size of 1.45 ms (the same analysis parameters used to compute the mel-spectrogram for the feature learning model). We summarized the MFCC coefficients over time using 11 summary statistics as in [48], and used the resulting feature vectors to train and test an SVM classifier with a radial basis function kernel.

**Hyper-parameters:** For the N-class scenario we evaluated the two models (baseline and proposed) using 5-fold cross validation. We determined model hyper-parameters separately for each fold by splitting the training set of the fold into training and validation subsets. We performed a grid search for model hyper-parameters by fitting the model to the training subset and evaluating it on the validation subset. After determining the best hyper-parameters, we fit the model to the full training set (of the fold) using the optimal hyper-parameter values, and then evaluated it on the fold’s test set. We repeated this process for every fold, and obtained the final accuracy score by taking the average over all 5 folds. For the acoustic monitoring scenario we split the data into train and test sets by season (details are provided in the Data section). Consequently, we determined model hyper-parameters by splitting the train set into train and validation subsets and performing the grid search as described above.

For the proposed feature learning approach, the hyper-parameters included the duration of the TF-patches used for feature learning *d*_patch_, the dictionary size *k*, the set of summary statistics *f*_stat_, and the penalty parameter *C* of the SVM. The values included in the grid search were:

- *d*_patch_ = {1.45, 5.8, 11.6, 23.2, 46.4, 92.9, 185.8} milliseconds.
- *k* = {128, 256, 512, 1024, 2048}
- *f*_stat_ = {{*mean*, *std*}, {*max*}, {*mean*, *std*, *max*}}, giving set sizes of *l* = {2, 1, 3} respectively.
- *C* = {1.0, 10.0, 100.0, 1000.0}.

For the baseline approach the only hyper-parameter was *C* (for which we evaluated the same 4 values listed above).

**Metrics:** For the N-class problem we evaluated model performance in terms of classification accuracy. Since the classes in the dataset used to evaluate this scenario (CLO-43SD, see the Data section for a detailed description) were not balanced, we also computed the per-class classification accuracy and the confusion matrix (which shows how many clips from each class were mistakenly classified and what they were classified as). To evaluate the sensitivity of the proposed model to every hyper-parameter, we gathered all the intermediate classification scores produced by the grid search for optimal hyper-parameter values conducted as part of the cross-validation process described earlier (there were 2100 such scores). We then grouped the scores, first by the value of *d*_patch_, then by the value of *k*, *f*_stat_ and *C*. Each grouping was used to produce a box plot that allowed us to compare the performance of the model as a function of every tested hyper-parameter value.

Unlike the N-class problem which is a multi-class classification task, the acoustic monitoring scenario is a binary classification task (i.e. every clip was classified as either containing a flight call from the species we were targeting or not). Given that each of the two datasets used to evaluate the acoustic monitoring scenario (CLO-WTSP and CLO-SWTH, see the Data section for a detailed description) was comprised of two highly-imbalanced classes, classification accuracy was not an appropriate metric. An alternative approach to evaluating binary models is to compute the Receiver Operating Characteristic (ROC curve) and the Area Under the (ROC) Curve (AUC) [49]. The ROC curve visualizes the trade-off between true positives and false positives (the closer the curve to the top-left corner the better), and the AUC summarizes the curve as a single number between 0 (worst) and 1 (best). A limitation of the ROC curve is that the difference observed between curves (as quantified by the AUC) is influenced by the degree of class imbalance. This means that, although it allows us to observe a difference in performance, for highly imbalanced sets (such as CLO-SWTH) it is hard to assess the degree to which model performance differs or the implications of this difference. A good model should detect most of the true target-species calls (high recall) and reject the vast majority of non-target clips (high precision). The latter (i.e. high precision) is particularly important, since a model that produces too many false positives cannot be used to reliably estimate the pattern of species occurrences over time. In light of this, another (potentially more informative) way to evaluate a binary model on unbalanced data is in terms of precision and recall as summarized by a Precision-Recall Curve (PR-curve). The PR-curve displays the model’s precision and recall for multiple possible threshold values between 0 and 1 (where the threshold is applied to the likelihood value produced by the model for each clip in order to make a classification decision). To provide as complete an evaluation as possible, we computed all of the aforementioned evaluation metrics that are relevant to the acoustic monitoring scenario: the confusion matrix, ROC curve, AUC, and PR-curve.

Finally, to assess the influence of background noise on the performance of the proposed model, we estimated the signal-to-noise ratio (SNR) between the flight call of the target species and the background noise for every positive clip (i.e. the clips that contained a flight call by the target species) in each of the two datasets (CLO-WTSP and CLO-SWTH). For each clip, we approximated the SNR by computing the average energy of the signal in the middle 150 ms of the clip where the flight call was found *P*_call_, and the average energy of the signal everywhere else in the clip *P*_background_ (each clip was roughly 1 s long, see the Data section for further details). Since the features used by our model only considered the spectrum between 2000 and 11025 Hz, we computed the spectral energy from the same frequency band. The approximate SNR is then given by:

$${\text{SNR}}_{\text{dB}}=10\log_{10}\left(\frac{P_{\text{call}}}{P_{\text{background}}}\right).$$ (5)

We divided the target clips into two groups based on the predictions made by the proposed model: the first group contained the true positives (correctly classified target clips) and the second contained the false negatives (incorrectly classified target clips). We then compared the SNR distributions of the two groups to see whether there was any relationship between SNR and model performance. We also tested to see whether there was a correlation between the approximate SNR and the confidence value returned by the proposed model for each clip. We obtained the confidence value, i.e. the probability that a clip belonged to the target class, by applying Platt scaling to the distance of the clip from the SVM’s separation hyper-plane [50].

## Data

### 43 Species Dataset (CLO-43SD)

To evaluate the classification model under the N-class scenario we compiled a dataset comprised of 5428 audio clips of flight calls from 43 different species of North American wood-warblers (in the family Parulidae), henceforth referred to as CLO-43SD. The clips came from a variety of recording conditions, including clean recordings obtained using highly-directional shotgun microphones, recordings obtained from noisier field recordings using omnidirectional microphones, and recordings obtained from birds in captivity using the method described in [51]. Every clip in this dataset was trimmed to contain a single flight call from one of the 43 target species A list of the species included in this dataset and the number of clips per species is provided in S1 Table.

### Fall 2014—Spring 2015 Migration Datasets

**Acoustic sensing:** To evaluate the model under conditions that realistically match the nocturnal migration monitoring scenario, we collected data using a prototype acoustic sensing system developed and deployed by the Cornell Lab of Ornithology. Two main components compose this system: a set of remote acoustic sensors and a centralized data server. Each acoustic sensor, or Recording and Observing Bird Identification Node (ROBIN), has a microphone, processor, and wireless Internet connection, and monitors the acoustic environment continuously. The sensor is equipped with a pressure zone microphone with a Knowles EK23132 microphone element. Each microphone is vertically oriented and its element sits at the bottom of a small plastic horn-shaped enclosure, which in turn sits inside a plastic housing. This configuration provides a doubling of sound pressure in the vicinity of the microphone element, where it sits near the hard plastic boundary of the horn enclosure. The external hard plastic enclosure aids in rejecting interfering sounds on the horizontal axis. The frequency response of this element is flat above 2 kHz, well suited to monitoring flight calls.

The ROBIN is powered by a commercially available Raspberry Pi Model B single-board computer (https://www.raspberrypi.org/products/model-b/) running the Cornell Lab of Ornithology’s own Realtime Acoustic Detection daemon (RADd). This daemon allows the processor to configure and to run a detection engine of one or more detectors simultaneously, with classification occurring on the server side and not on the Raspberry Pi. Currently the RADd is running template detectors. Each data template detector uses representative calls to serve as templates [52]. Correlation scores are calculated by using spectrogram cross correlation to compare each template to the audio stream, resulting in a score between 0 and 1. When the score goes above a defined threshold, the detector is activated and an audio clip of roughly 1 second in duration centered on the detected event is recorded and transmitted to the data server over WiFi for classification. The detected audio clips are stored in the data server alongside metadata such as the sensor location, the date and time each clip was recorded, and the species-specific detector that triggered and recorded the clip.

For this study each sensor was loaded with two detectors, one for each of two species: White-throated Sparrow (WTSP) and Swainson’s Thrush (SWTH). Since the goal of these detectors is to maximize the recall of flight calls from the target species, they were set with a relatively low threshold (0.6), meaning each detector returned thousands to hundreds of thousands of clips over a single migration season (between 2–4 month), where the vast majority of those clips did not contain any flight call, and the remainder were comprised of calls by the target species and calls from other (non-relevant) species. Our goal was to train the classification model to perform a binary classification of each clip, where the two possible classes were the target species (WTSP or SWTH) and “everything else”, which henceforth shall be referred to as “other”. Consequently, we trained a separate (binary) model for each of the two species.

**Data collection:** Two sensors were deployed in the fall of 2014, one in Ithaca, NY (ITH) and another in New York, NY (NYC). 8 more sensors were deployed in the spring of 2015, giving a total of 10 sensors: one in NYC and 9 in up-state NY. The sensors monitored the environment and transmitted audio clips of potential flight call detections over a total period of 6 months: from September to December 2014 (4 months) and from April to May 2015 (2 month). The former represents all or almost all of the fall 2014 migration season for WTSP and SWTH, and the latter all or almost all of the spring 2015 migration season. In the fall, WTSP detectors transmitted 5016 clips, all from the NYC location. SWTH detectors transmitted 8666 clips, the majority (8248) from the NYC location, and the rest from ITH. In the spring WTSP detectors transmitted a total of 11687 clips (n.b. from all 10 sensors) and SWTH detectors transmitted 170445 clips (also from all 10 sensors). For both species, we used the fall 2014 clips for training, and the spring 2015 clips for testing. Since practically all of the training data came from the NYC sensor, while the test data came from all 10 stations, our training and testing sets were mismatched both in location and season. This situation provides a challenging classification task that requires the model to generalize well from the training conditions to the test conditions.

One of us (AF) manually annotated every 1 sec detection clip, each of which came from a species-specific detector (e.g. WTSP), with “WTSP”, “Flight Call” (for flight calls other than the target species) or “Reject” (for non-flight calls) labels. We maintained these categories, but we also combined the latter two groups into an “Other” category for our experiments. The annotation process to label and review labels of flight calls requires significant expertise with flight calls, including knowledge of the diversity of inter- and infraspecific variation and the different ways this variation can be expressed in aural and visual forms of review. With over three decades of experience with flight call identification from field and laboratory perspectives, including classification by ear (i.e. by listening) and by eye (i.e. reviewing spectrograms), we are confident in our annotations. Additionally, during our error analysis (cf. Results and Discussion) we found no classification errors resulting from human confusion among call identities. Furthermore, although we found a small numbers of labeling errors in each of our review processes, all of these were clerical in nature. After three rounds of reviewing the calls and their labels, we found our data to be error free.

Most flight calls have a duration of less than 150 ms, and training a model on a full 1 second clip, at least 850 ms of which might not contain relevant data, could result in most training data not containing the relevant signal (the flight call). To account for this, we trimmed automatically every clip to 150 ms, centering on the middle of the original file with the assumption that a flight call was most likely to be centered around the precise time at which the template detector was triggered. In a random inspection of a small subset of 100 clips from the dataset, we found that all inspected clips still contained the flight call after the trimming procedure.

For the remainder of this paper we treat the aforementioned data as two separate datasets: one containing all the fall and spring clips returned by the WTSP detectors, and the other containing all the fall and spring clips returned by the SWTH detectors (CLO-WTSP and CLO-SWTH, respectively). A summary of the two datasets with train/test (fall/spring) splits is provided in Table 1. The “%Pos” column shows the percentage of positive instances in each data subset. Note that in both datasets the number of false positives returned by the template detectors (the sum of FlightCall and Reject) is considerably greater than the number of true positives (Target), as evident from the low positive instance percentages. This scenario is extreme in the case of SWTH clips during spring 2015, where the template detectors returned only 316 true positives compared to over 170000 false positives. The task of correctly identifying true positives while rejecting all false positives is, therefore, extremely challenging. To ensure reproducibility, the two datasets (CLO-WTSP and CLO-SWTH) as well as the CLO-43SD dataset are available online (http://dx.doi.org/10.5061/dryad.j2t92).

**Table 1:** Summary of the two datasets used to evaluate the model under the acoustic monitoring scenario: CLO-WTSP and CLO-SWTH. For each dataset a breakdown is provided into train data, test data, and total. For each breakdown we provide the number of instances for the positive class (Target) and for the negative class, where the latter is further divided into negative instances containing flight calls other than the target species (FlightCall) and negative instances containing no flight call (Reject). The percentage of positive instances in each set is provided in the %Pos column.

Dataset	Train (fall 2014)				Test (spring 2015)				Total (fall + spring)			
	Target	FlightCall	Reject	%Pos	Target	FlightCall	Reject	%Pos	Target	FlightCall	Reject	%Pos
CLO-WTSP	882	1063	3071	21.3%	656	1569	9462	5.9%	1538	2632	12533	10.1%
CLO-SWTH	876	806	6984	11.2%	316	197	169932	0.2%	1192	1003	176916	0.7%

## Results and Discussion

### N-class problem with CLO-43SD

**Fig 2:**
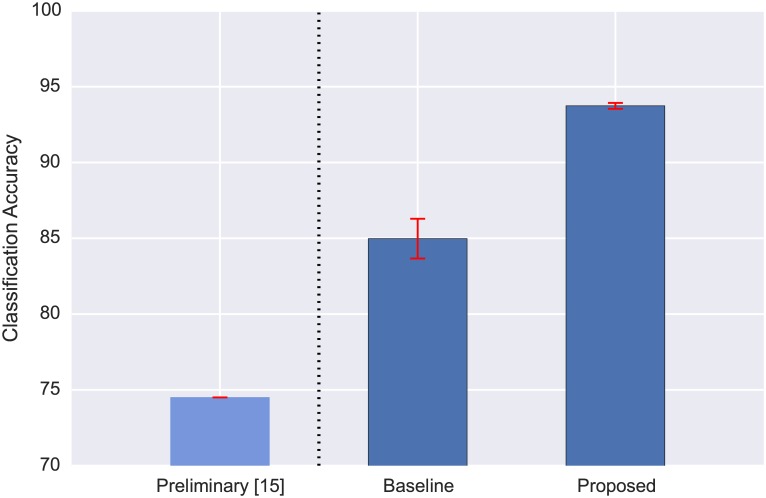
Classification accuracy of the proposed model for the N-class problem using CLO-43SD. The proposed model is compared against a baseline method which uses standard MFCC features. For additional context the preliminary result reported in [15] for a flight call dataset with a similar number of species (42) is also provided, however it is not directly comparable to the baseline and proposed model since the study used a smaller dataset of 1180 samples. The error bars represent the standard deviation over the per-fold accuracies (for [15] there is only a single value).

**Fig 3:**
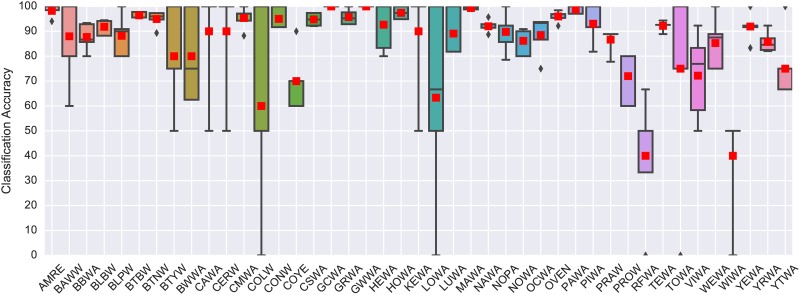
Per-class (per-species) classification accuracy obtained by the proposed model for each of the 43 species in CLO-43SD. The box plots are derived using 5-fold cross validation, where the red squares represent the mean score for each species. A mapping between the abbreviations used in this plot and the full species names is provided in S1 Table.

We begin with the results obtained for the N-class problem. The classification accuracy yielded by the baseline approach and the proposed model are presented in Fig 2. The proposed model performed well on the N-class problem, obtaining an average classification accuracy of 93.96% and significantly outperforming the MFCC baseline (84.98%) as determined by a two-sample Kolmogorov-Smirnov test (statistic = 1.0, p-value = 0.003, sample size = 5 (folds)). Since the classes in CLO-43SD were not balanced, we also computed the per-class accuracies (Fig 3) and the confusion matrix for all 5 folds combined (S1 Fig). Despite the class imbalance, the model yielded near or above 90% accuracy for the majority of species (the average per-class accuracy was 86%), with only 4 species going below 70%, which is understandable given that there were only 13 or fewer instances of those classes in the dataset. The confusion matrix is very sparse, indicating the model rarely made mistakes, and the few notable confusions can be attributed to the low number of instances of the confused classes in the dataset.

The results confirm that the learning-based model is suitable for flight call classification, even for a relatively high number of classes (43 species), and complement the results obtained for birdsong classification in [20]. Whether this performance translated to the bioacoustic monitoring “species versus other” scenario was one of the key questions of this study, and will be answered in the following section.

**Fig 4:**
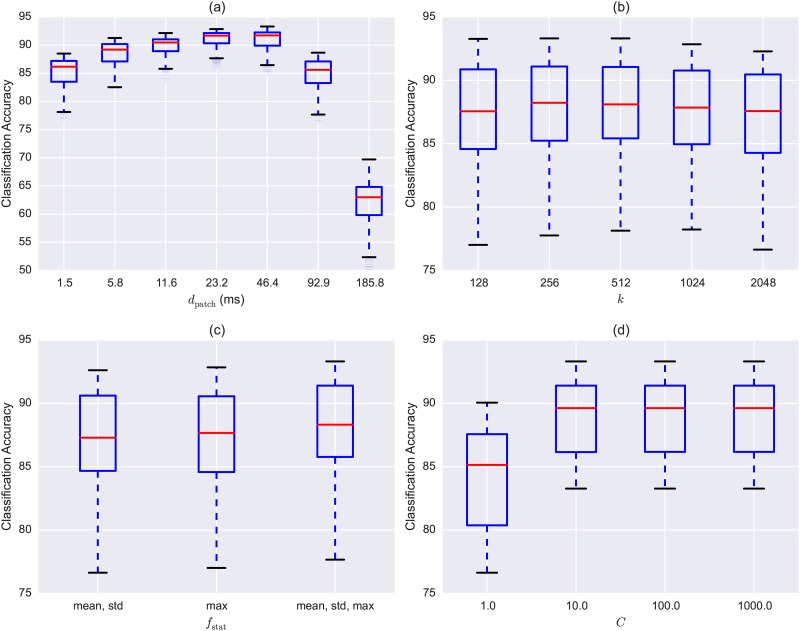
Model sensitivity to hyper-parameter values for CLO-43SD. Each subplot displays the classification accuracy as a function of: (a) the duration of the TF-patches *d*_patch_, (b) the size of the codebook *k*, (c) the set of summary statistics used in feature encoding *f*_stat_, and (d) the penalty parameter *C* used for training the Support Vector Machine classifier.

Finally, we explored the sensitivity of the model to each hyper-parameter, displayed in Fig 4. The most influential parameter was *d*_patch_ (the duration of the TF-patch): using longer patches (thus learning larger spectro-temporal structures) increased accuracy up to a patch duration of 46.4 ms. Beyond that the patch duration spans most or all of the flight call, and this proved to be detrimental to the model under this scenario. Interestingly, using a small dictionary size of 128 was sufficient, and increasing *k* did not result in improved accuracy. This result stands in contrast to that observed for urban sounds in [34], possibly due to the reduced variance in flight calls of the same species compared to more heterogeneous sounds such as sirens or jackhammers which have more diverse sound production mechanisms and patterns. The model was also relatively robust to the choice of summary statistic and *C* value (for *C* > = 10).

### Acoustic monitoring with CLO-WTSP

Next we turn to the nocturnal migration monitoring scenario, starting with the CLO-WTSP dataset. Recall that the training set contained 882 WTSP clips and 4134 “other”, and the test set contained 656 WTSP clips and 11031 “other”. The hyper-parameter values used for *d*_patch_, *k*, *f*_stat_ and *C* (determined via cross-validation on the training set) were 92.9 ms (64 frames), 128, {*max*} and 1.0 respectively. The optimal hyper-parameters values were notably different compared to the N-class scenario: here an even longer TF-patch duration of 92.9 ms proved to be beneficial, while the optimal *C* value was 1. This tells us that the model must be parameterized differently for the N-class and the acoustic monitoring scenarios and is the first (but not last) indication in this study that one should not expect results obtained for the former scenario to generalize to the latter.

As noted in the Methods section, classification accuracy was not a relevant metric for this scenario (the class imbalance meant that even a model that labeled everything as “other” would yield an accuracy of 94.4% on the test set). Instead, we directly examined the confusion matrix (Table 2), which shows that the model was successful at rejecting noise clips (only 6 false positives), but it also rejected most of the real WTSP flight calls.

**Table 2:** Confusion matrix yielded by the proposed model for the CLO-WTSP test set. Row labels represent the true class and column labels represent the class predicted by the model.

	**WTSP**	**OTHER**
**WTSP**	40	616
**OTHER**	6	11025

**Fig 5:**
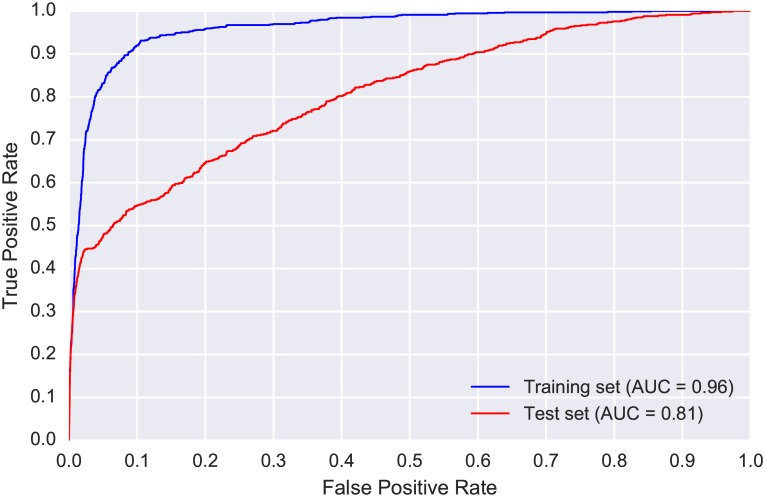
Receiver Operating Characteristic (ROC) curves produced by the proposed model for CLO-WTSP: training set (blue, obtained via 5-fold cross validation) and test set (red). The Area Under the Curve (AUC) score for each set is provided in the figure legend at the bottom right corner.

**Fig 6:**
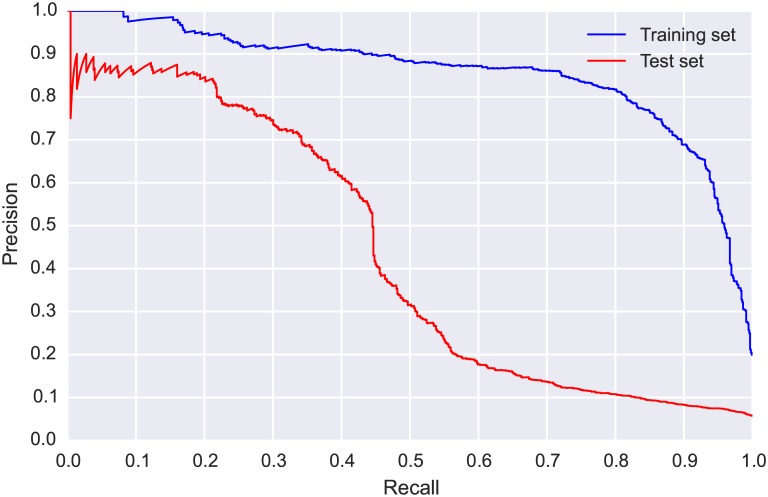
Precision-recall (PR) curves for CLO-WTSP: training set (blue, obtained via 5-fold cross validation) and test set (red).

The ROC curve and AUC for the train and test sets are presented in Fig 5. We observed a drop in performance between the training and test sets, highlighting the challenge presented by this real-world scenario in which we must cope with mismatched conditions between the training and test sets (different location, different time of year). Further insight can be gained by examining the PR-curves for the train and test sets, presented in Fig 6. The difference in performance is more pronounced in the PR-curves, and allows us to make direct performance observations: the model could detect WTSP calls in the test set with relatively high precision (a low number of false positives) for recall values up to 0.4. For greater recall values (obtained by using more relaxed threshold values) the precision dropped markedly and the model became considerably less reliable.

To gain further insight into the limitations of the model, we conducted a detailed error analysis in which one of us (AF) examined (visually and aurally) every misclassified clip (as determined by the confusion matrix in Table 2). Starting with the false negatives, we identified three primary potential causes for model confusion: of the 616 wrongly rejected clips, 42% (258) included some other loud source (such as insects or wind noise) that was at least partially masking the flight call. 82% (504) contained a range of unusual variants of more typical WTSP flight calls, with observable (visual and aural) variation in the calls’ fundamental frequency trajectories compared to more expected patterns. Differences included varying numbers of frequency peaks, varying slope of descending frequency contour at the start of the call, and varying modulation to end the call, among other features. Such calls are unlikely to be well represented in the training set, particularly given the suite of variants composing these more unusual calls, resulting in their misclassification during testing (suggestions for dealing with this problem are provided further down). Finally, 95% of the false-negative clips (588) contained a call that was either distant or quiet compared to the acoustic environment. 31% had both interference and an unusual call, 37% interference and were quiet, 78% were quiet and unusual, and 30% included all three factors. This analysis highlighted that the main challenges faced by the model for this dataset were background noise, low signal energy and limited training data.

**Fig 7:**
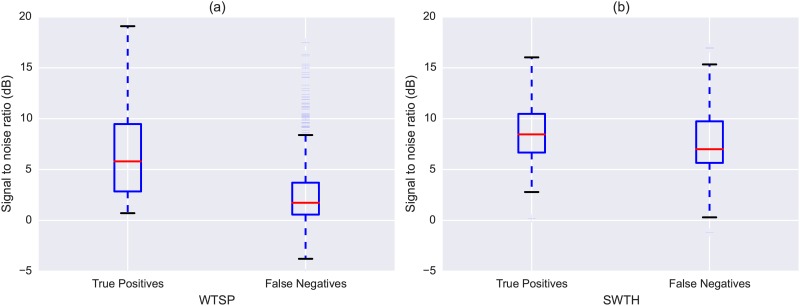
Approximate Signal-to-Noise-Ratio (SNR) computed separately for the true positives and false negatives returned by the proposed model: (a) CLO-WTSP test set, (b) CLO-SWTH test set.

In Fig 7(a) we compare the SNR values for the WTSP true positives and false negatives. There is a clear difference between the two sets, with true positives having better (higher) SNR values than false negatives. This difference is statistically significant as determined by a two-sample Kolmogorov-Smirnov test (statistic = 0.44, p-value = 4.7 × 10^−7^, sample sizes of 40 and 616 for true positives and false negatives respectively), and provides quantitative confirmation of our observations based on the qualitative error analysis presented earlier. As explained in the Methods section, we also tested whether there is a correlation between the approximate SNR and the confidence value returned by the SVM classifier. Indeed, we found the two to be positively correlated (Pearson correlation coefficient of 0.37, p-value = 1.3 × 10^−23^, degrees of freedom (df) = 654), meaning there was a tendency for the model to produce more confident predictions the greater the SNR of the flight call was compared to the background.

Dealing with unusual calls could be addressed by increasing our training set in future iterations, making sure to include as many of these unusual calls as possible so that the classifier can better model the variance of the target class. An alternative (and more scalable) solution is to use *data augmentation* techniques to generate a variety of modulated calls from the existing training data. Such techniques have shown great success in improving model generalization [53], including for audio classification problems [54]. Finally, we examined the 6 false positives, and all clips did actually contain a WTSP call: 2 were labeling errors, and AF rejected the other 4 because they occurred during diurnal recording periods.

### Acoustic monitoring with CLO-SWTH

Next, we repeated the analysis for the CLO-SWTH dataset. As noted earlier, this dataset was considerably more challenging due to the extremely disproportionate ratio between the positive class (316 SWTH calls) and the negative class (170129 “other” clips). This time, the hyper-parameter values selected for *d*_patch_, *k*, *f*_stat_ and *C* were 23.2 ms (16 frames), 512, {*mean*, *std*} and 1.0 respectively. This shows that not only does the optimal model parametrization change from the N-class to the acoustic monitoring scenario—it also changes within the acoustic monitoring scenario depending on the specific species we are trying to detect.

**Fig 8:**
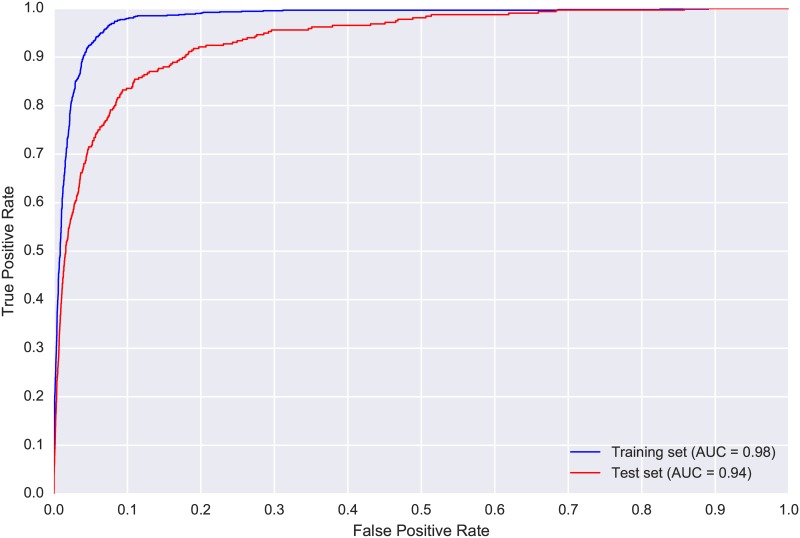
Receiver Operating Characteristic (ROC) curves produced by the proposed model for CLO-SWTH: training set (blue) and test set (red). The Area Under the Curve (AUC) score for each set is provided in the figure legend at the bottom right corner.

**Fig 9:**
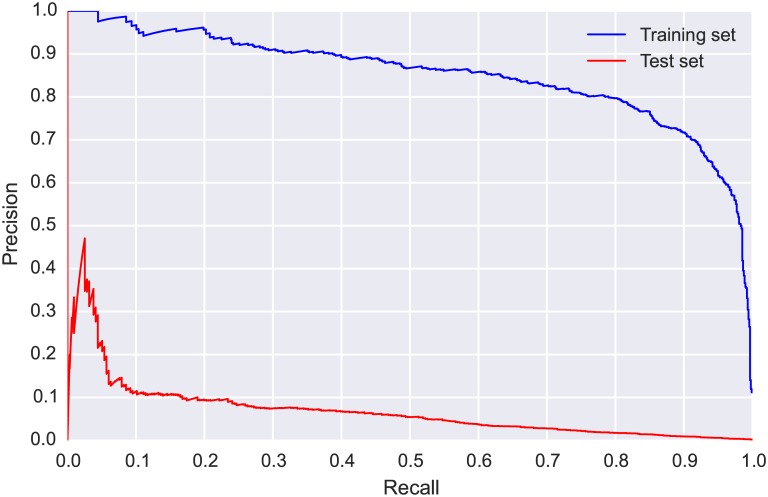
Precision-recall (PR) curves for CLO-SWTH: training set (blue, obtained via 5-fold cross validation) and test set (red).

The confusion matrix (Table 3) shows that even though the model correctly identified more than half of the true SWTH flight calls and rejected over 160000 noise clips, it still generated over 5000 false positives. The considerable class imbalance means the ROC curves and AUC values (Fig 8) are not really informative for this dataset, and we must examine the PR-curves (Fig 9) to gain meaningful insight. The PR-curve for the test set shows that, even with a very strict threshold, the precision never goes above 0.5, and with such a threshold the model would retrieve less than 5% of the true SWTH calls. This suggests that, unlike for WTSP, for SWTH there is no threshold value for which the model would produce satisfactory results on the test set.

**Table 3:** Confusion matrix yielded by the proposed model for the CLO-SWTH test set. Row labels represent the true class and column labels represent the class predicted by the model.

	**SWTH**	**OTHER**
**SWTH**	194	122
**OTHER**	5396	164733

To gain further insight we conducted the same error analysis for SWTH as we did for WTSP. Starting with the 122 false negatives reported in Table 3, 76% (93) contained interference, 55% (68) contained an unusual call, and 60% (74) contained calls that were distant or quiet. 42% (51) contained interference and an unusual call, 43% (52) interference and a quiet call, 33% (40) an unusual and quiet call, and finally 23% (28) of the clips included all three factors. From Fig 7(b) we see the difference in approximate SNR between true positives and false negatives is not as notable as it was for WTSP, though still statistically significant (KS statistic = 0.23, p-value = 0.0005, sample sizes of 194 and 122 for true positives and false negatives respectively). However, we found only a weak correlation between the SNR and model confidence (Pearson correlation coefficient of 0.13, p-value = 0.018, df = 312).

Unlike WTSP, for SWTH the model returned a considerable number of false positives (5396). Upon examination, we discovered that the vast majority (5261 or 97%) were clips containing vocalizations of spring peepers (*Psuedscris cruficier*), a frog species that produces a sound remarkably similar, aurally and visually when inspecting the spectrogram, to the SWTH flight call. This problem could be addressed in several ways in future studies: first, we could discard springtime detections from ROBIN units at locations known to contain peepers, though this is not ideal nor scalable. Second, we could work on improving the microphone technology on the sensors such that they only capture sound coming from above the sensor, for example through the use of microphone arrays and beamforming [55, 56]. From a machine listening perspective, the obvious solution is to include peeper samples in our training set—the training set used for the current model included flight calls from non-target bird species, but not from other taxonomic groups (e.g. Class Amphibia) such as, in this case, frogs.

### Flight call detection histograms

**Fig 10:**
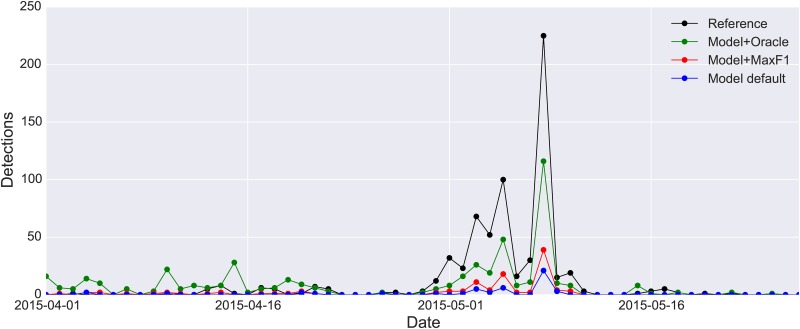
Detection curves showing the daily number of detected WTSP calls in the CLO-WTSP test set. The true curve (the reference, computed from the expert annotations) is plotted in black. The other three curves represent detections generated by the proposed model using different threshold values: the default (0.5) in blue, the threshold that maximizes the f1 score (which quantifies the trade-off between precision and recall by computing their harmonic mean) on the training set (0.33) in red, and the “oracle threshold” (0.11) that maximizes the f1 score on the test set in green.

**Fig 11:**
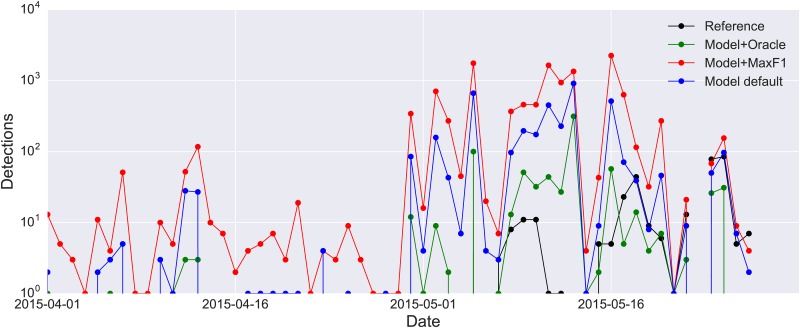
Detection curves showing the daily number of detected SWTH calls in the CLO-SWTH test set. The true curve (the reference, computed from the expert annotations) is plotted in black. The other three curves represent detections generated by the proposed model using different threshold values: the default (0.5) in blue, the threshold that maximizes the f1 score (which quantifies the trade-off between precision and recall by computing their harmonic mean) on the training set (0.29) in red, and the “oracle threshold” (0.73) that maximizes the f1 score on the test set in green.

Given the reduced detection precision of the model in the acoustic monitoring scenario as evidenced by the PR-curves obtained for WTSP and SWTH, we must ask: is the model precise enough to reliably identify the pattern of species occurrences over time? Furthermore, what threshold value should we use (i.e. on the likelihoods produced by the model) to decide which clips should be labeled as positive detections? To answer this, we plotted the detection results once more, this time as a histogram of daily detections over the 2-month migration period. The results are presented in Fig 10 for WTSP and Fig 11 for SWTH (note the log-scaled y-axis in the latter).

When examining the detection curves for WTSP (Fig 10) we see that both the default detection curve (blue) and the curve obtained by maximizing the f1 score on the training set (red) are fairly conservative. The oracle curve is closer to the reference curve during the peak of the migration season, but also produces more false positives during April. What is encouraging about these curves though is that they are all highly correlated with the reference curve. The Pearson correlation coefficient between the red curve (which is the best we could do in a real-world scenario) and the reference curve is 0.98 with a p-value of 1.7 × 10^−40^ (df = 55). In fact, it is more strongly correlated to the reference curve than both the default curve (0.95) and the oracle curve (0.93). So although we cannot trust the absolute counts generated by our model, we can use the model to determine the migration *pattern* (or trend) for WTSP; this result is highly promising.

Finally, we turn to the detection curves obtained for SWTH. Given the poor PR-curve obtained by the model for the CLO-SWTH test set, it is unsurprising that all three detection curves (default, optimal f1 and oracle, Fig 11) contain a large amount of false detections on various days (note the log-scaled y-axis). Based on our qualitative error analysis, we know the vast majority of these false detections are triggered by spring peepers. Indeed, the model produces so many false positives on certain days that on a linear scale the reference curve in black appears almost flat in comparison. Consequently, the detection curves are uncorrelated to the reference curve (Pearson correlation coefficient of 0.014, -0.002 and 0.065 respectively). The negative result for SWTH highlights two important points: first, even a model that correctly rejects the vast majority of non-target clips (approximately 165000 of 170000) might be too imprecise to produce a reliable detection curve. For the N-class problem such a high classification accuracy (96%) would be considered satisfactory in most cases, further highlighting the increased complexity of the continuous monitoring scenario. It also clearly demonstrates that a model that performs well on the former might not translate to the latter, in this case due to the abundance of a previously unseen confounding source (the spring peeper), a problem that cannot be identified in the N-class (closed-set) setting where all the data are known in advance. Second, it is evident that the task of the classifier has been made excessively challenging by the poor performance of the SWTH template detector running on the remote sensors, producing hundreds of thousands of false positives. Consequently, in addition to continued work on the model itself, we must dedicate time to developing a better detector. Due to the relatively low processing power of the remote sensors, the challenge lies in producing a detector that performs better than the simple spectral cross-correlation approach, whilst being computationally efficient. To this end, we are currently investigating the performance of onset detection algorithms [57] that are efficient enough to run in real-time. Another option would be to develop more complex classification models for generic flight-call detection and reduce their complexity via model compression [58] so that they can be run on limited devices such as the Raspberry Pi.

## Conclusions and Future Work

In this paper we investigated the automatic classification of bird species from their flight calls. In particular, we examined and contrasted the differences between two classification scenarios: the N-class problem and the continuous acoustic monitoring scenario, which is more relevant to automatic nocturnal migration monitoring via bioacoustic analysis. To do so, we implemented a state-of-the-art audio classification model based on unsupervised feature learning. We evaluated the model on three novel datasets, one for studying the N-class problem including over 5000 flight calls from 43 different species, and two realistic datasets for studying the nocturnal migration monitoring scenario that were compiled by means of remote acoustic sensors deployed in the field during the fall 2014 and spring 2015 migration seasons. By examining the performance of the model on these datasets we identified the challenges presented by the continuous monitoring scenario and contrasted them to the more controlled N-class classification problem. We showed that although the model performs very well on the N-class problem, it is not equally successful for the bioacoustic monitoring scenario. Furthermore, we showed that the model must be optimized differently for each scenario and differently for each species in the acoustic monitoring scenario. In the case of one species (White-throated Sparrow) we observed that, despite a decrease in performance, the model was nonetheless able to produce a detection curve that is highly correlated to the reference. This type of bioacoustic information complements existing sources of information about bird migration, in particular from Doppler weather surveillance radar [59] and eBird [8, 60]. Radar data can describe the density, direction, speed, and altitude of migrating birds at night, but they cannot describe the species involved; eBird data describe the location, number, and identity of species, but almost exclusively ground-based observations during diurnal periods. Data from bioacoustic migration monitoring, such as the detection curve produced by the model for WTSP, describe species composition at night while migrating birds are actually migrating, providing a critical link between the taxonomically agnostic radar data and temporally mismatched eBird data. The model did not produce an equally reliable curve for Swainson’s Thrush patterns. Interestingly, the main source of confusion for the model was not other bird species, but the presence of spring peepers, a species of frog that produces a vocalization remarkably similar to the SWTH flight call. In addition to continued work on the model itself, template detectors running on the acoustic sensors must be replaced with an algorithm that produces less false positives but is still computationally efficient (i.e. due to the sensors’ limited processing power).

Also, while we can compute the precision of the template detectors from the clips they produce (i.e. the ratio of true positives to false positives), we cannot currently evaluate their recall rate. That is, since the sensors only return clips when a detection is triggered, we have no way of knowing whether the template detectors are missing any true positives. As part of our future work we will collect continuous recordings of full nights (8–10 hours each) at sensor locations during a migration season. This will allow us to compare clips returned by the detectors to the true number of flight calls emitted by the target species during these nights and compute the detector recall rate. We already noted that the precision of the current detectors, especially the SWTH detector, is too low and that we must employ an alternative approach that produces fewer false positives. At the same time, we must ensure that the detection technique we deploy on the sensors has a very high recall rate, since any flight calls not returned by the detectors cannot be recovered at the latter stage of specific species classification using a more complex model running on our server.

The results of this study suggest a number of future directions for research. As already noted, we need to develop a high-recall low-complexity detector with better precision than the current template detectors to run on the remote sensors. In this context, we intend to investigate adaptive noise filtering techniques [16] to reduce the amount of false positives returned by the detectors. Such filtering could also potentially improve the performance of our species classification by reducing the variance of the background conditions the model has to deal with and improving its generalizability from one environment to another. Still, it is clear that even with noise filtering the detector is bound to produce a large amount of false positives, and so we must also work on improving the performance of our species classification method. Given that the proposed feature learning approach outperformed the baseline technique which uses a standard feature set, it is reasonable to assume that a deeper learning architecture such as the one employed by convolutional neural networks [61–65] might perform better still, and we intend to investigate this set of classification architectures. Finally, given that some of the observed errors stem from the lack of representative data, we also plan to conduct extended data collection and annotation in combination with data augmentation techniques [53, 54] as part of our future work.

## Supporting Information

S1 Table Full name, abbreviation, and number of instances of each species in the CLO-43SD dataset. (PDF)

S1 Fig Confusion matrix for the proposed model on the CLO-43SD dataset (all 5 folds combined). Row labels represent the true class and column labels represent the class predicted by the model.
(PDF)
