# Supplementary material for: Towards the Automatic Classification of Avian Flight Calls for Bioacoustic Monitoring
Source: PLoS One. 2016 Nov 23;11(11):e0166866. doi: 10.1371/journal.pone.0166866 (PMC5120805; doi:10.1371/journal.pone.0166866)
Supplement: S1 Table — (PDF) [file pone.0166866.s001.pdf]

S1 Table. CLO-43SD Species List.

Table 1: **Full name, abbreviation, and number of instances of each species in the CLO-43SD dataset.**

Name	Abbreviation	Instances
Magnolia Warbler	MAWA	1256
Black-throated Green Warbler	BTNW	376
Nashville Warbler	NAWA	351
American Redstart	AMRE	336
Ovenbird	OVEN	323
Tennessee Warbler	TEWA	269
Black-throated Blue Warbler	BTBW	224
Grace's Warbler	GRWA	212
Yellow-rumped Warbler	YRWA	196
Hooded Warbler	HOWA	193
Chestnut-sided Warbler	CSWA	191
Cape May Warbler	CMWA	172
Palm Warbler	PAWA	170
Blackburnian Warbler	BLBW	86
Orange-crowned Warbler	OCWA	78
Bay-breasted Warbler	BBWA	73
Northern Parula	NOPA	67
Yellow Warbler	YEWA	63
Virginia's Warbler	VIWA	60
Connecticut Warbler	CONW	59
Pine Warbler	PIWA	58
Lucy's Warbler	LUWA	55
Blackpoll Warbler	BLPW	52
Northern Waterthrush	NOWA	51
Common Yellowthroat	COYE	50
Prairie Warbler	PRAW	45
Worm-eating Warbler	WEWA	42
Blue-winged Warbler	BWWA	40
Golden-winged Warbler	GWWA	33
Golden-cheeked Warbler	GCWA	29
Hermit Warbler	HEWA	29
Prothonotary Warbler	PROW	25
Black-and-white Warbler	BAWW	24
Black-throated Gray Warbler	BTYW	22
Cerulean Warbler	CERW	18
Townsend's Warbler	TOWA	18
Yellow-throated Warbler	YTWA	16
Red-faced Warbler	RFWA	13
Canada Warbler	CAWA	12
Louisiana Waterthrush	LOWA	11
Wilson's Warbler	WIWA	10
Colima Warbler	COLW	10
Kentucky Warbler	KEWA	10
