# Supplementary material for: Towards the Automatic Classification of Avian Flight Calls for Bioacoustic Monitoring
Source: PLoS One. 2016 Nov 23;11(11):e0166866. doi: 10.1371/journal.pone.0166866 (PMC5120805; doi:10.1371/journal.pone.0166866)

S1 Fig. Confusion matrix for the proposed model on the CLO-43SD dataset (all 5 folds combined). Row labels represent the true class and column labels represent the class predicted by the model.

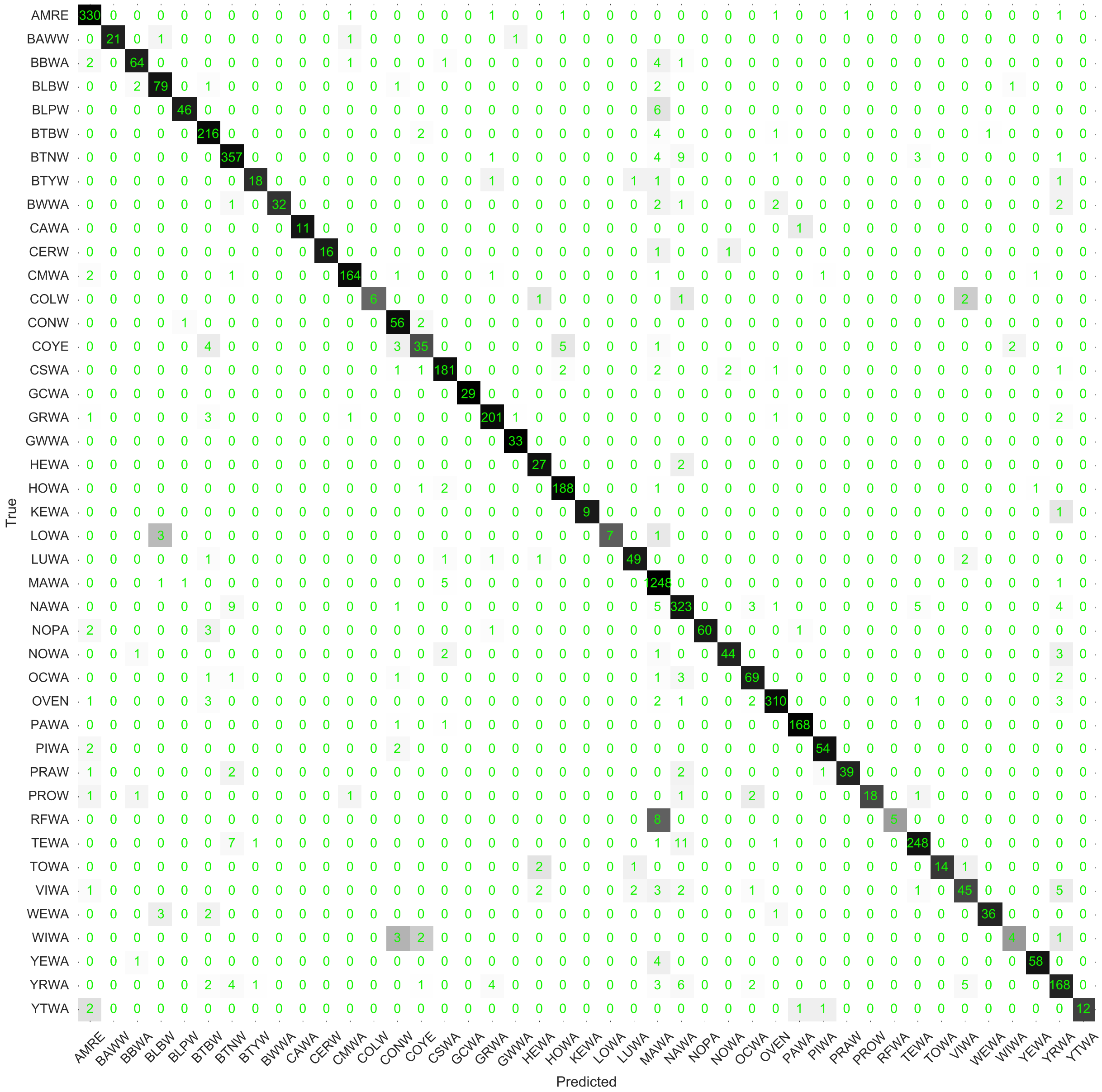

Supplement: S1 Fig — Row labels represent the true class and column labels represent the class predicted by the model. (PDF) [file pone.0166866.s002.pdf]
